# Camera-Aided Orientation of Mobile Lidar Point Clouds Acquired from an Uncrewed Water Vehicle

**DOI:** 10.3390/s23136009

**Published:** 2023-06-28

**Authors:** Hannes Sardemann, Robert Blaskow, Hans-Gerd Maas

**Affiliations:** Institute of Photogrammetry and Remote Sensing, Technische Universität Dresden, 01062 Dresden, Germany; hannes.sardemann@tu-dresden.de (H.S.);

**Keywords:** mobile lidar, multisensor platform, riverbank mappings, uncrewed water vehicle

## Abstract

This article presents a system for recording 3D point clouds of riverbanks with a mobile lidar mounted on an uncrewed water vehicle. The focus is on the orientation of the platform and the lidar sensor. Rivers are areas where the conditions for highly accurate GNSS can be sub-optimal due to multipath effects from the water and shadowing effects by bridges, steep valleys, trees, or other objects at the riverbanks. Furthermore, a small measurement platform may have an effect on the accuracy of orientations measured by an IMU; for instance, caused by electromagnetic fields emitted by the boat rotors, the lidar, and other hardware decreasing IMU accuracy. As an alternative, we use exterior orientation parameters obtained by photogrammetric methods from the images of a camera on the boat capturing the riverbanks in time-lapse mode. Using control points and tie points on the riverbanks enables georeferenced position and orientation determination from the image data, which can then be used to transform the lidar data into a global coordinate system. The main influences on the accuracy of the camera orientations are the distance to the riverbanks, the size of the banks, and the amount of vegetation on them. Moreover, the quality of the camera orientation-based lidar point cloud also depends on the time synchronization of camera and lidar. The paper describes the data processing steps for the geometric lidar–camera integration and delivers a validation of the accuracy potential. For quality assessment of a point cloud acquired with the described method, a comparison with terrestrial laser scanning has been carried out.

## 1. Introduction

Survey vessels are a useful and efficient tool to record water bodies and their surrounding shore and bank areas. Small, shallow, hazardous, or restricted waters may require the use of small uncrewed water vehicles (UWVs). In addition to their application in the ocean [1], uncrewed surface vehicles (USVs), as they are also called, can be applied in inland waters. One application is the acquisition of three-dimensional (3D) point clouds and models of the morphology of small rivers where crewed survey vessels are oversized and conventional surveying methods are too time consuming. Furthermore, UWVs allow for automatic data acquisitions.

### 1.1. Uncrewed Water Vehicles as Multisensor Platforms

Equipping crewed or uncrewed vessels with multiple sensors enables the acquisition of high-resolution 3D point clouds. Here, a distinction can be made between the measurements of above- and underwater geometries. Echo sounders are commonly used to record river bathymetry. While larger rivers allow for survey vessels with multibeam echo sounders [2], smaller rivers require echo sounders mounted on UWVs [3]. Optical methods enable depth measurements in even shallower areas [4]. Mapping above the water level can be realized by cameras and lidar systems. Terrestrial laser scanners operated in profiler mode can be mounted on larger platforms, like crewed motorboats. In [5], such a system is used to capture the shoreline of two lakes. Compact lidars can be mounted on UWVs as well. In [6] a combination of a camera and a Velodyne Puck lidar is used to detect objects on the water in a marine environment.

Besides oceans and lakes, rivers are a possible area of application for multisensor water vehicles. Ref. [7] shows that the modeling of hydrological processes demands new measurement methods, especially at low cost for worldwide applications. High-resolution 3D models of the river morphology can be helpful to improve the prediction and understanding of flood events. Analyzing the impact of a flood event on the riverbanks helps to gain a better understanding of the process. Especially when rivers are located in forested areas, riverbanks are often difficult to measure with camera-equipped airborne systems such as unattended aerial vehicles (UAVs) due to shadowing effects. Water vehicles may operate under the trees and are able to capture the riverbanks with optical sensors at close range. UWVs are a low-cost alternative to large survey vessels that enable a broad application not only in small rivers. In Ref. [8] a multisensor dataset containing data of different waterways recorded with a lidar system, stereo cameras, GNSS, IMU, and radar mounted on a UWV is presented.

### 1.2. Camera-Based Orientation

To generate a 3D point cloud from a mobile lidar on a moving platform, position and orientation of the sensor have to be known for every captured 3D lidar point. The most common method to determine the orientation of an outdoor operated platform is the combined use of data from a Global Navigation Satellite System (GNSS) receiver and an inertial measurement unit (IMU) [9]. In this case, the orientation of the platform is calculated using the IMU’s high temporal resolution acceleration and rotation rate measurements. Due to the drift vulnerability of an IMU, and for georeferencing, the trajectory calculation by the IMU is supported by GNSS measurements that are usually available in a lower temporal frequency. Shading caused by high vegetation surrounding the rivers or high rock formations may lead to an interruption of the GNSS measurement. In cases where the GNSS signal is not available or erroneous and where no (expensive) high-quality IMU is available, the pose of a multisensor platform may be also derived from photogrammetric multi-image triangulation.

Simultaneous localization and mapping (SLAM) algorithms provide poses in real time. Using SLAM with camera images or video image sequences is often referred to as visual odometry [10]. In Ref. [11] a recent review of visual SLAM algorithms is given. SLAM has the disadvantage that it tends to drift when the path is not closed (dead reckoning). Particularly in river mapping, it is often favorable to steer the boat in only one direction, which is mostly downstream, leading to an open path. When real time is not needed, structure from motion (SfM) techniques may be utilized for 3D point cloud and camera orientation determination in post processing. Herein, using control points enables georeferenced and scaled 3D point clouds as well as sensor orientation parameters that do not suffer from drifts. Ref. [12] presentes a georeferencing procedure for a moving platform, integrating camera and lidar observations.

The aforementioned methods give the position and orientation of a mobile platform referring to the coordinate system of the camera that was used for the orientation determination. However, lidar points are recorded in a scanner coordinate system defined by the lidar. When both sensors are fixed on the platform, a relative orientation between scanner and camera coordinate system can be calibrated in order to determine the orientation of the lidar. Existing methods often use planar objects for the calibration of the relative orientation. In Ref. [13] a checkerboard pattern is moved through the object space to calibrate the extrinsic calibration of a camera and a line scan lidar. In Ref. [14] there is also a planar chessboard pattern used for the extrinsic calibration of a 16-channel 3D lidar and a system of six cameras. These methods require the manual interaction of a user moving the plane. In Ref. [15] a 3D calibration set-up consisting of multiple boxes is utilized. This method only needs one viewpoint for the calibration once the calibration field is established. The resulting accuracies are in the range of centimeters to decimeters for the position determination.

### 1.3. Outline and Innovations of This Article

This article analyzes the quality of camera-based position and orientation determination for a lightweight lidar system. A method will be presented that can be used for the georeferencing of a mobile lidar point cloud when GNSS and IMU poses are not available or are erroneous. This is particularly important for UWVs navigated on small rivers. The method relies on a camera that is operated in video or time-lapse mode, a mobile lidar, control points on the riverbanks, and a low- to mid-cost IMU to bridge very short time periods. First, the basic concept of the camera-based lidar sensor orientation is presented (Section 2). Then, the strategy for the underlying calibration of the geometric relative orientation as well as time-sync between scanner and camera is described (Section 3). The geometric calibration process (Section 3.1) only requires the acquisition of an image and a lidar scan. Once the calibration field is established and measured, it can be used for a quick and mm-accurate geometric calibration of a mobile mapping camera-lidar system. Subsequently, a kinematic calibration process for the temporal synchronization of lidar and camera is presented in Section 3.2. A calibrated system enables the transformation of lidar points, which is described in Section 4. For the evaluation of the presented method, it has been applied for a UWV-based measurement of a riverbank (Section 5). The resulting point cloud is compared to reference measurements that were generated with a terrestrial laser scanner (TLS) (Section 6). The paper ends with a conclusion and suggestions for future research (Section 7).

## 2. Platform Orientation Determination

In order to reference and merge 3D points recorded with a mobile lidar, the orientation of the scanner has to be known. It is, therefore, important to know the current position and orientation of the platform during the entire measurement. The common choice to determine the position and orientation of a mobile platform (outdoors) is to use differential GNSS for the position and IMU for the orientation. IMU measurements on a UWV are highly affected by electromagnetic fields emitted from the scanner, the boat rotors, and other electrical equipment on the platform. The most prominent effect of that can be observed in the heading. Furthermore, GNSS is influenced by multipath effects on the water surface and shadowing by objects on the riverbanks.

**Figure 1:**
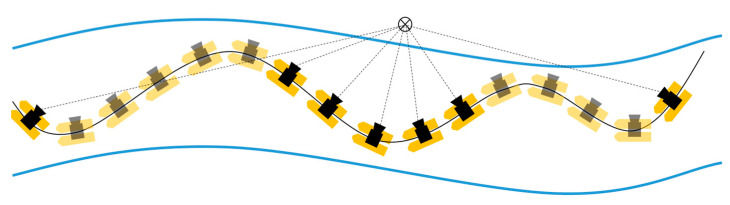
UWV trajectory. A zig-zag trajectory enables the visibility of object points from various distances and directions.

This article, therefore, evaluates the quality of camera-based orientations for platform orientation determination. Processing time-lapse images of a camera in an SfM procedure including control points results in georeferenced positions and orientations for all images referring to the pose of the camera coordinate system (ccs). Thus, the camera trajectory in the world coordinate system (wcs) can be derived from the image data. For every image i, there is a six-parameter transformation matrix, Mccsiwcs(ω,ϕ,κ,X,Y,Z), containing its exterior orientation using homogeneous coordinate transformations. In order to acquire convergent image observations, a zig-zag trajectory should be applied (Figure 1). Each object point is then seen in multiple images from multiple directions. The interior orientation of the camera can be determined with self-calibration in the same process.

## 3. Calibration of Lidar to Camera Orientation

The exterior orientations of a UWV can be used to register the lidar frames and to combine them to a point cloud of the riverbanks. The platform orientations based on the camera images define the position and orientation of the camera coordinate system in world coordinates. The 3D points measured by the lidar are recorded in the scanners own coordinate system (scs). In order to transform lidar points from scs to wcs, using the orientations from Section 2, the relative orientation between scs and ccs has to be calibrated. This calibration consists of two steps: a geometric calibration of boresight alignment and lever arm, and a time synchronization between the camera and the lidar clock.

### 3.1. Geometric Calibration

**Figure 2:**
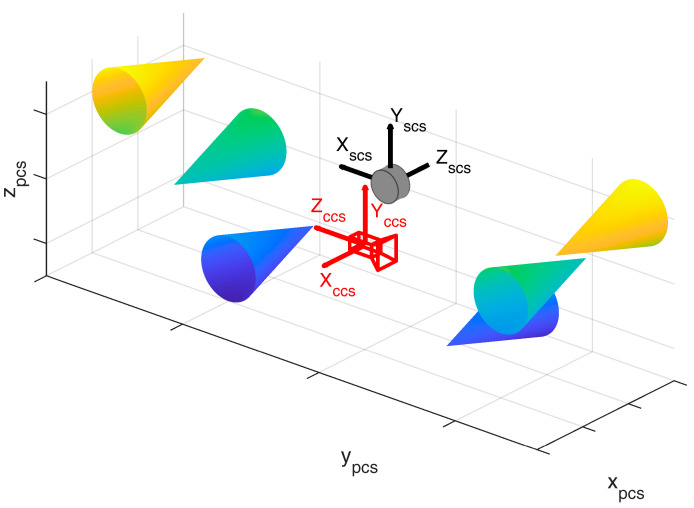
Calibration field set-up. Camera (red) and lidar (grey) both observe the cone-based calibration field that is located in a project coordinate system (pcs). Six cones are placed on two sides of the scanner at different distances and heights in order to determine the relative orientation between the camera coordinate system (*ccs*) and the scanner coordinate system (*scs*). The cones are color coded according to the height.

The geometric calibration process is an improved version of the method presented in [16]. Therein, a cone-based calibration procedure was used for the intrinsic calibration of a 2D laser scanner, while we use a similar method for the calibration of relative orientations between scs and ccs, which was not part of the method in [16]. It consists of several cones placed at different distances and heights in the field of view of both the lidar and the camera (Figure 2). The geometry of the calibration field has to be known with high accuracy, resulting in exact positions and orientations of the cones in a project coordinate system (pcs).

**Figure 3:**
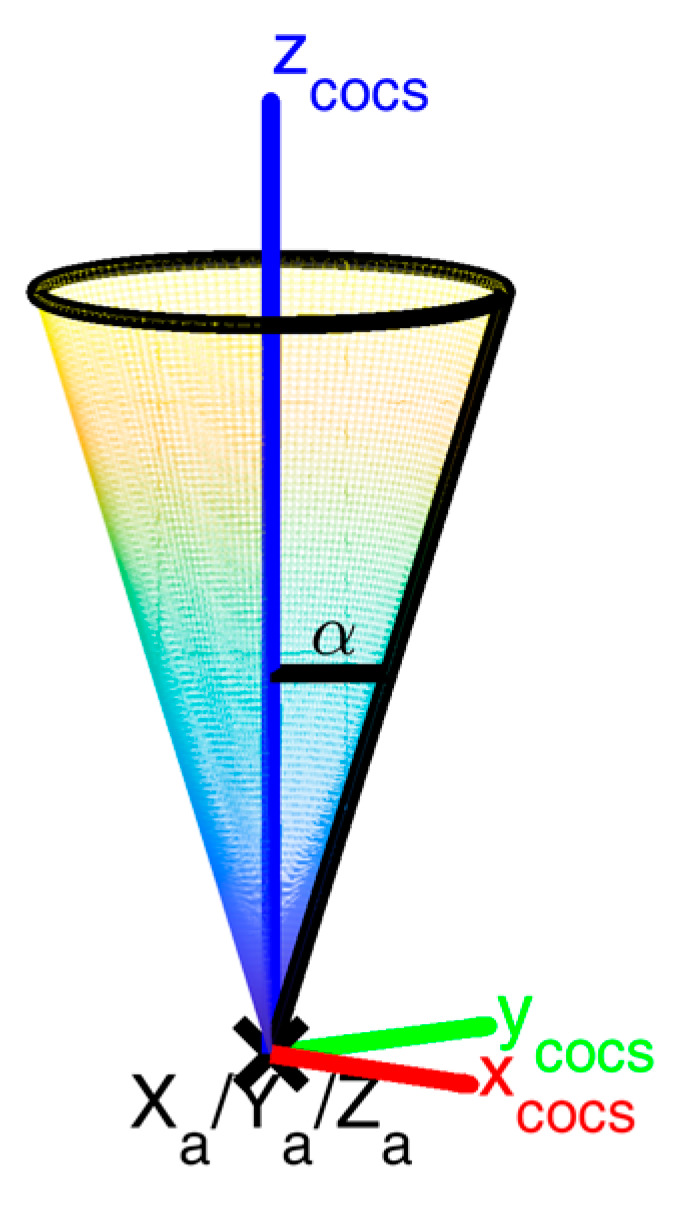
Cone coordinate system. Each cone of the calibration field has its own coordinate system (cocs) defined by its apex coordinates ($x_{a},y_{a},z_{a}$), the *z*-axis along the cone’s axis, and an opening angle α.

Each cone has its own cone coordinate system (cocs), with the origin in its apex a and the *z*-axis along the cone’s axis (Figure 3). Points $p_{j}$ on the surface of a cone can be transformed into cone coordinates by a translation with the apex coordinates and a rotation from pcs to cocs (Rpcscocs). The rotation includes only two angles (λ,θ), since the cone is rotation invariant:

(1) [xyz]pjcocs=Rpcscocs(λ,θ)⋅([xyz]pjpcs−[xyz]pcsa)

All cone points fulfil the condition

(2) (xpjcocs)2+(ypjcocs)2−(zpjcocs)2tan2(α)=0,

where α is the opening angle of the cone.

**Figure 4:**
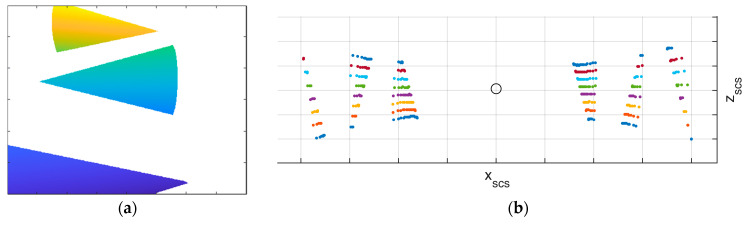
(**a**) Synthetic camera image of the cone-based calibration field, where the color of the cones indicates height. (**b**) Synthetic lidar frame of the cone-based calibration field, where the color indicates the scanline ID of a lidar with eight scanlines with 4° steps. The black circle indicates the lidar position.

A camera image and a 3D point cloud frame are recorded for at least one static position. The cones are visible both in the lidar frames and in the images. Figure 4 shows the synthetic camera image and lidar frame recorded from the positions depicted in Figure 3.

The exterior orientation Mccsipcs of the camera image can be determined in project coordinates using spatial resection. Furthermore, the individual cones have to be cropped from the scanner frame and used for an orientation determination of the lidar point clouds. This can be achieved in a common least squares optimization with the model from Equations (1) and (2). Equation (2) is applied for the reference points (in pcs) of all six cones and simultaneously for the lidar points (in scs) of the same cones. For the lidar points, the model has to be extended with a transformation from scanner into project coordinates before they can be fitted with the same cone parameters. The lidar orientation in project coordinates is determined relative to the camera using the relative orientation matrix Mscsccs, which has to be determined in the optimization process:

(3) [xyz1]pcs=Mccsipcs⋅Mscsccs⋅[xyz1]scs

The total number of parameters is six per cone (cone parameters) and six for the relative orientation (Mscsccs), totaling 42 parameters when six cones are used. While the calibration strategy is suitable to be used with only one single recording, it can still be extended with more positions for a better accuracy and liability.

### 3.2. Time Synchronization

Mobile lidars can usually be synchronized with GPS time. Some cameras on the other hand, especially customer cameras, do not support external triggering. In this case, indirect time synchronization has to be applied. For that purpose, the calibration process is extended by a second step. In addition to the static positions that were used for the relative geometric orientation determination in the previous section, a dynamic acquisition is performed. The UWV is moved along the calibration field and images are recorded in video or time-lapse mode. Exterior orientations are calculated for all of those images as well. At the same time, RTK positions have to be recorded with a GNSS receiver on board the UWV. In order to find the offset between GPS time and camera clock, the GNSS and camera positions need to be available in the same world coordinate system, wcs. The calibration field, therefore, has to be georeferenced. For every image observation time $T_{i}$ (in GPS time), the GNSS antenna is located at an offset (dx,dy,dz) in camera coordinates:

(4) [dxdydz]ccsTi=Mccsi−1wcs⋅[XYZ]GNSSTi wcs

However, images are not recorded with a GPS timestamp $T_{i}$, but with a camera timestamp $t_{i}$. The temporal offset dt between thee camera time and the GPS time can be defined by T=t+dt. Substituting and rearranging Equation (4) leads to an equation with four unknowns (dt,dx,dy,dz) that can be solved in a Gauss–Helmert optimization:

(5) Mccsi−1wcs⋅[XYZ]GNSS(ti+dt) wcs−[dxdydz]ccs=0

## 4. Lidar Point Transformation

Given that the relative orientation and temporal synchronization of the camera and the scanner enables the transformation of 3D lidar points using the respective image orientation, a 3D point then has to be transformed from the scanner coordinate system to the image coordinate system and from image coordinates to world coordinates using Equation (3). The orientation of one image is used for the registration of one lidar frame. A lidar frame is hereby defined as a full 360° rotation of the lidar centered on the image recording time with half of the rotation before and the other half after the image was taken. Since the platform is not still during the acquisition of one lidar frame, using the same camera-based pose for all points of one frame would lead to an error in the georeferenced point cloud. Thus, the pose used for the orientation of each lidar point is interpolated using the IMU. Therefore, relative orientations between the IMU and camera orientation are determined for all image timestamps and interpolated for the lidar-point timestamps. The missing orientations between the image acquisition times can then be derived from the IMU measurements by applying the interpolated relative orientations.

## 5. Experiments

**Figure 5:**
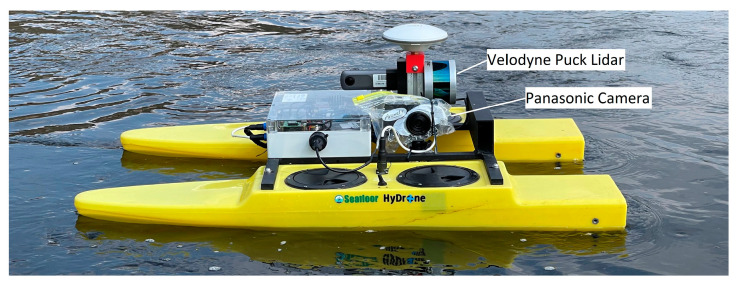
The uncrewed water vehicle used in this study. The Velodyne Puck lidar is attached vertically on the back, while the Panasonic camera is oriented towards the left in the direction of travel, protected with a soft plastic cover.

The methods presented in the previous paragraphs were tested with experimental data acquired with a UWV that was navigated along a river. The UWV used for this study is a Seafloor HyDrone (Figure 5). It is equipped with a two-frequency GNSS receiver (Swiftnav Piksi Multi) and an IMU (Advanced Navigation Spatial) for position and orientation determination. Riverbanks are observed with a mobile lidar (Velodyne Puck). The Velodyne Puck is a very popular lidar sensor in low- or mid-cost mobile mapping systems, offering the advantages of a good price–performance ratio and easy integration. It records 500,000 points per second in 16 scanlines with horizontal and vertical fields of view of 360° and 30°, respectively. It has a maximum distance of 100 m and a 3D point accuracy of 3 cm. The UWV was first presented in [17] in an earlier stage of development. It may also be equipped with an underwater laser triangulation sensor [18]. The sensor platform is designed to be modular so that different sensors can be attached according to the measurement task.

For this study, a Panasonic DMX-GX80 camera with 15.8 megapixels and a 14 mm lens was attached to the UWV for camera-based orientation determination and to capture high-resolution images of the riverbank. The camera was chosen because of an available time-lapse mode, but could be replaced by other models as well. It can be installed on either side of the UWV according to the riverbank of interest. The camera was used in time-lapse mode with an image acquisition rate of 1 Hz.

**Figure 6:**
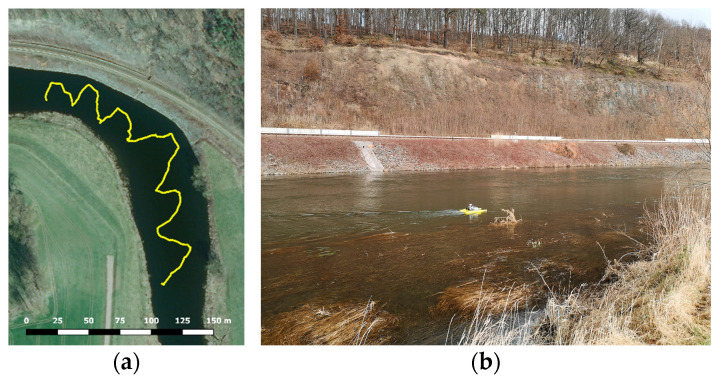
The Freiberger Mulde. (**a**) GPS-track of the transect. (**b**) UWV on the river with the railroad bank in the background.

The UWV was applied on the river Freiberger Mulde in Germany to map the riverbanks. The acquisition was undertaken in only one transect, where the platform was steered downstream in a zig-zag pattern from one riverside to the other (Figure 6a). The measurement took 17 min steering with mean speeds of 0.4 m/s (lateral) and 7°/s (angular). The right-hand riverbank is the area of interest being a sloped railroad embankment with large stones and small vegetation like bushes and small trees (Figure 6b). The river had a width of approx. 40 m at that location and time of measurement.

### 5.1. Reference Point Cloud

**Figure 7:**
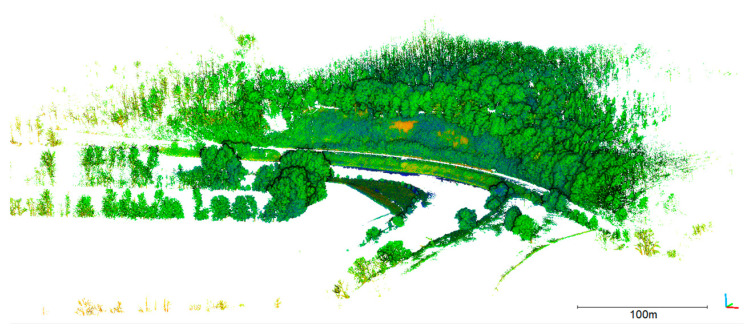
Terrestrial laser scan of the Freiberger Mulde (reflectance colorized).

A reference point cloud has been recorded with terrestrial laser scanning using a Riegl VZ400i (Figure 7). Three stations have been recorded and merged. Georeferencing was realized with circular targets that were measured with RTK GNSS. The point cloud has a mean point spacing of 2.5 cm. The 3D point accuracy specified by the manufacturer is 3 mm at 50 m distance.

### 5.2. Calibration and Synchronization Results

The calibration process from Section 4 was performed before the measurement. The relative orientation between the scanner and the camera was determined from three viewpoints where the UWV was placed in the middle of the calibration field. Since time synchronization was solved in a subsequent step, static positions were needed to assign the camera image and lidar frame. The positions differ mostly in orientation. Coded markers were attached to the surface of the cones and the geometry of the calibration field was measured with superior accuracy: First, only the marker coordinates were determined in a bundle block adjustment using Aicon 3D Studio. Including additional scale bars with known length allowed for object point accuracies of <1 mm herein. The 3D coordinates and their corresponding image measurements were imported into Agisoft Metashape, where a dense point cloud was determined. Furthermore, for time synchronization, the reference point cloud of the calibration field was georeferenced using circular targets that were measured with RTK.

**Figure 8:**
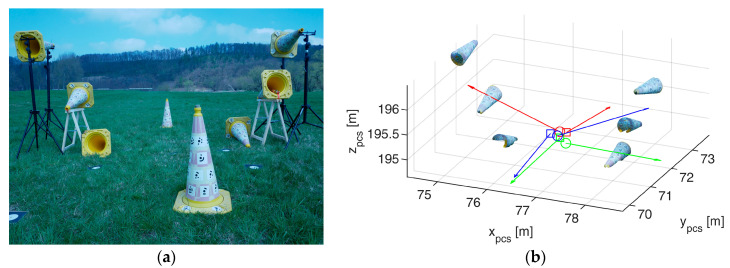
Geometric calibration. (**a**) Calibration field consisting of 8 cones, from which only the 6 horizontal ones were used for calibration. (**b**) Camera and scanner positions determined using the cones from the dense point cloud. Circles show camera positions and squares show scanner positions. The arrows give the orientation of the *z*-axis. Same color indicates same UWV position.

The exterior orientation parameters of the three Panasonic camera images were determined in the same SfM project, with interior orientation being calibrated in advance. The corresponding Velodyne positions were estimated relative to the image orientations using Equations (1)–(3) in an optimization process (Figure 8). The lever arm between lidar and camera has a length of 16 cm and has been determined with a standard deviation of <1 mm. The relative orientation angles have standard deviations of <1 mrad. The exact values can be found in Table 1.

**Figure 9:**
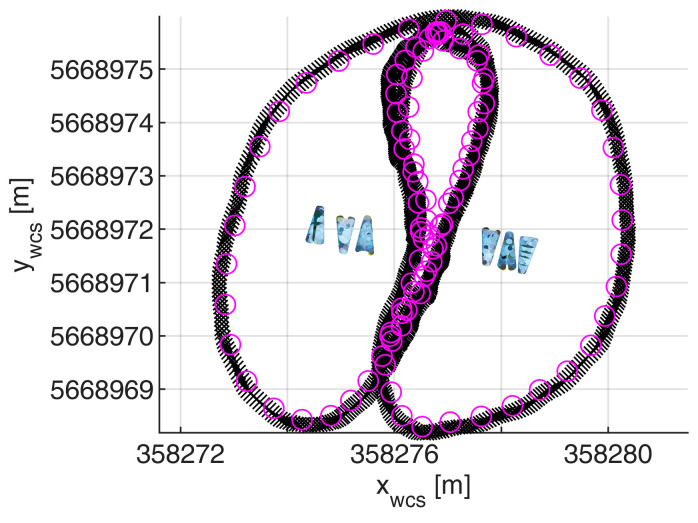
Time offset calibration. Image positions are shown as pink circles while the GNSS track is shown as black x.

The same calibration field was used for the time synchronization of the camera clock and GPS time. For that purpose, the UWV was moved around the calibration field with images taken every second. The images have been oriented in the SfM project. An RTK track of the boat-based GNSS antenna has been recorded at the same time with a frequency of 10 Hz. Figure 9 shows both tracks. Equation (5) was used to determine the temporal and spatial offsets between both tracks. The calibrated time offset shows a standard deviation of 0.004 s.

### 5.3. Transformation of Mobile Lidar Point Clouds

**Figure 10:**
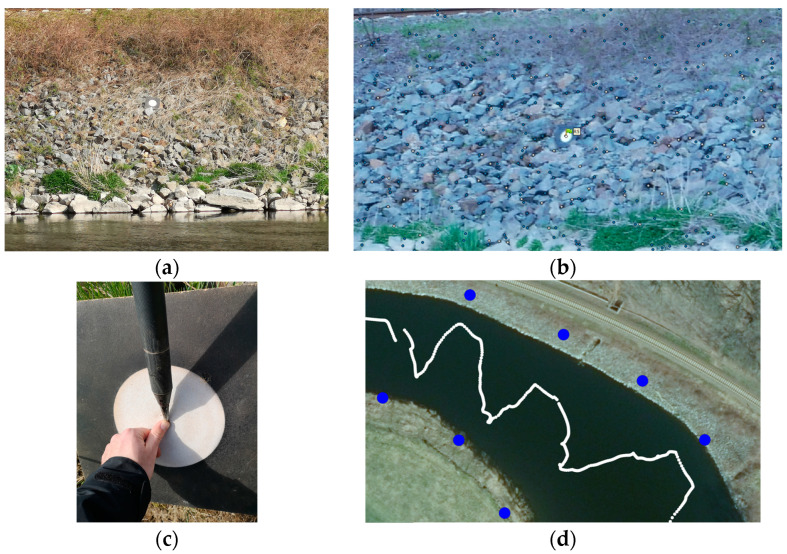
Image Orientation determination and georeferencing. (**a**) Control point on river-bank. (**b**) Image measurement in Metashape. (**c**) RTK measurement. (**d**) Camera locations (white circles) and control point locations (blue circles) on aerial image.

Images were gathered every second along the track shown in Figure 6a and were aligned in Agisoft Metashape, using control points that were measured with RTK (Figure 10).

**Figure 11:**
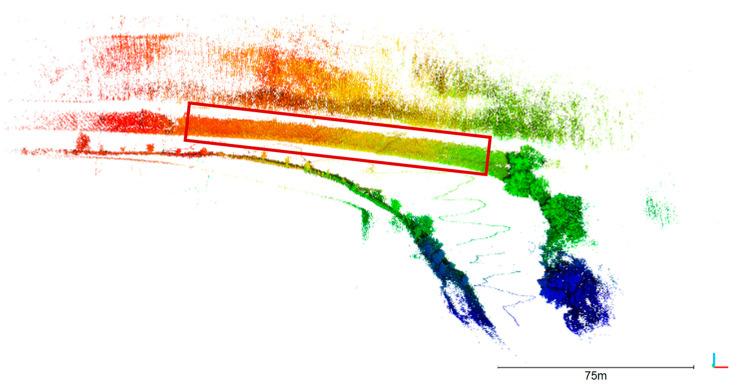
Oriented mobile lidar point cloud. The color indicates the image number that was used for transformation of the lidar frame. The red box shows the area of interest that was used for further analysis.

The image orientations and the calibrated relative orientation between the camera and the lidar were used to calculate 3D-world-coordinates of every lidar point using Equation (3). Figure 11 shows the resulting point cloud generated from 1025 lidar frames at the corresponding image positions.

The outside bank is of specific interest since it is subject to erosion. The area of interest (red box in Figure 11) was clipped from the point cloud and used for further analysis. Due to the zig-zag trajectory, most parts of that area have been measured from more than one UWV position, resulting in a variation of measurement distances: 99% of all points were measured within 60 m distance.

## 6. Accuracy Analysis

The input parameters that were used to generate the resulting 3D point cloud (Figure 11) were determined with a certain accuracy. This leads to a point cloud, which includes a certain error. The following section treats aspects of error propagation to analyze the expected and achieved accuracy.

### 6.1. Theoretical Accuracy

The acquisition of a 3D point cloud from a mobile lidar operated on a UWV with orientation determination from images involves several error sources which affect the point cloud accuracy. The main error sources are the orientation of the platform, the time synchronization of lidar and camera, the relative orientation, and the lidar measurement itself. These individual error sources can be combined to an overall expected 3D point uncertainty using the law of error propagation.

#### 6.1.1. Platform Orientation Accuracy

**Figure 12:**
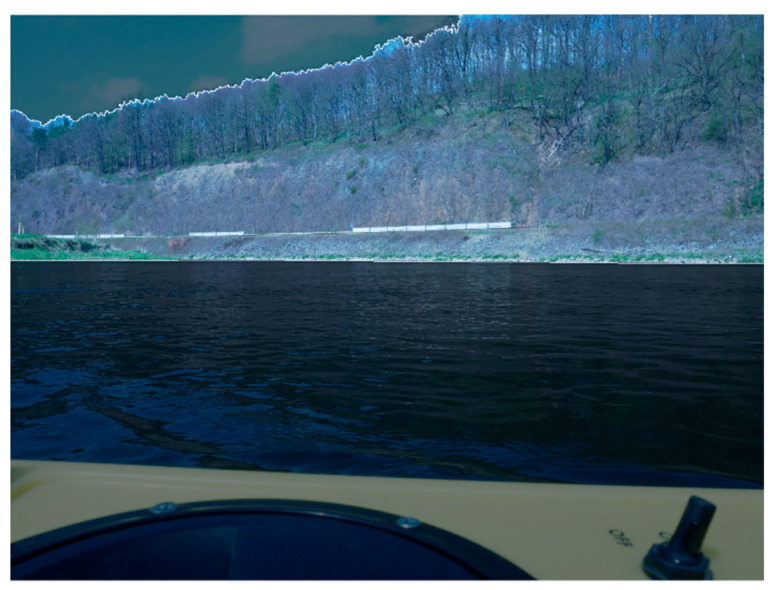
Measurement image used for SfM. Large areas of the image (sky and water) are not suited for matching and, therefore, masked out.

The accuracy of an exterior orientation determination with SfM depends on various factors like overlap of the images, geometry of ray intersections, or contrast in the images. Figure 12 highlights that a large portion of the images cannot be used for image matching: the UWV platform is visible in the image, being unsuited for matching. The water body shows reflections and movement and, therefore, results in mismatches. The sky shows moving clouds that cannot be used for matching either. These areas need to be masked out before calculation, leaving only a small area for matching and orientation determination. Masking has been conducted automatically using a ‘Masks From Color’ python script for Metashape. Overall, mean standard deviations of 5 mm for position and 0.004°–0.078° for the orientation parameters have been achieved (see Table 1).

#### 6.1.2. Time Synchronization Accuracy

**Figure 13:**
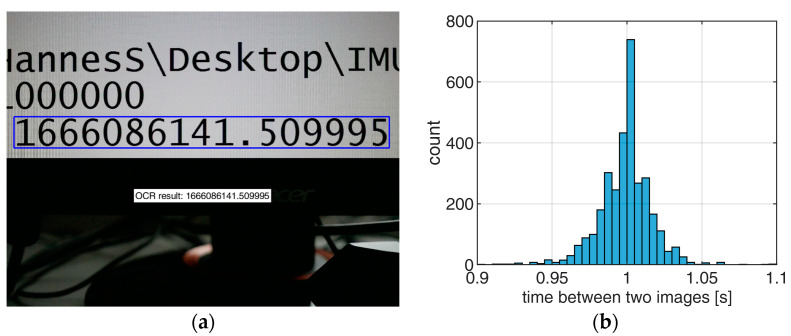
Determination of camera clock offset. (**a**) GPS time stamp on monitor. (**b**) Time between two images in time-lapse mode with 1 Hz.

The concepts of Section 3 assume that the interval between two images is exactly one second. A laboratory experiment was conducted to find the timing stability of the camera clock. An exact time stamp that was gathered by an IMU was, therefore, displayed on a computer monitor with a frequency of 100 Hz. The camera was placed in front of the monitor and images of the time stamp were captured in time-lapse mode with 1 Hz (Figure 13a). Optical character recognition was applied using the MatLab function ‘ocr’ to read the time stamp from each image for a period of approx. 90 min. The mean time between two images was 0.999 s with a standard deviation Δt of 0.023 s (Figure 13b).

The timing error results in wrong assignments between lidar points and camera orientations. Since the UWV is moving, Equation (3) should be extended to

(6) [XYZ1]wcs=(Mwcsccsi+δMccsiδt⋅Δt)⋅Mccsscs⋅[XYZ1]scs

where $\frac{\delta M_{ccs_{i}}}{\delta t}$ is the speed of the UWV in position and angle and Δt is the time synchronization error. A mean speed of 0.4 m/s (lateral) and 7°/s (angular) can be assumed (Section 6). The resulting error on the object world coordinates is mostly caused by the angular movement of the UWV during Δt and is listed in Table 1.

#### 6.1.3. Calibration Accuracy

The relative orientations between image and lidar coordinates can only be determined to a certain accuracy, as shown in Section 4. Their standard deviations and influences on the object coordinates are included in Table 1.

#### 6.1.4. Lidar 3D Point Accuracy

The Velodyne Puck user manual [19] lists a typical 3D point accuracy of 3 cm, independent of measurement distance. Ref. [20] shows that the accuracy in fact decreases with distance and is furthermore dependent on the material of the measured object. Since there is no accuracy analysis available for larger distances, this analysis will assume the manufacturer’s declaration. In order to obtain a standard deviation for all three coordinate components, the 3D error is split into three equal parts:

(7) sscsx/y/z =(sscs3D)23=17.3 mm

#### 6.1.5. Propagation of Errors

All individual error sources can be used to calculate an expected measurement 3D point error for the mobile lidar point cloud. Each 3D point is calculated using Equation (6). The expected error on the point cloud coordinates (X,Y,Z)wcs can be estimated using the law of error propagation. Assuming uncorrelated errors, the partial derivatives of Equation (6) with respect to each input variable are calculated and multiplied (squared) with their variance:

(8) swcsX/Y/Z=∑((δ(X/Y/Z)δvi)2⋅svi2)

where $v_{i}$ are the 16 input variables: (ω,ϕ,κ,x,y,z)wcsccs, Δt, (ω,ϕ,κ,x,y,z)ccsscs and (x,y,z)scs. Table 1 lists their standard deviations and estimated influences on the 3D accuracy of the world coordinates for four exemplary measurement distances:

(9) swcs3D=swcsx2+swcsy2+swcsz2

It is assumed that the three components of (X,Y,Z)scs are equal, which does not reflect the real measurement behavior of a Velodyne Puck, but simplifies the consideration of the 3D point errors.

The angular errors show, as expected, a higher influence on the overall measurement accuracy, especially in larger distances. The most dominant error source are the time synchronization errors (line 7 in Table 1), also originating from an angular movement of the UWV.

### 6.2. Experimental Results

**Figure 14:**
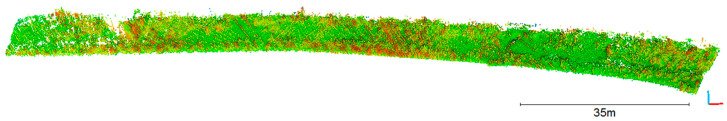
Mobile lidar point cloud to TLS mesh distance.

**Figure 15:**
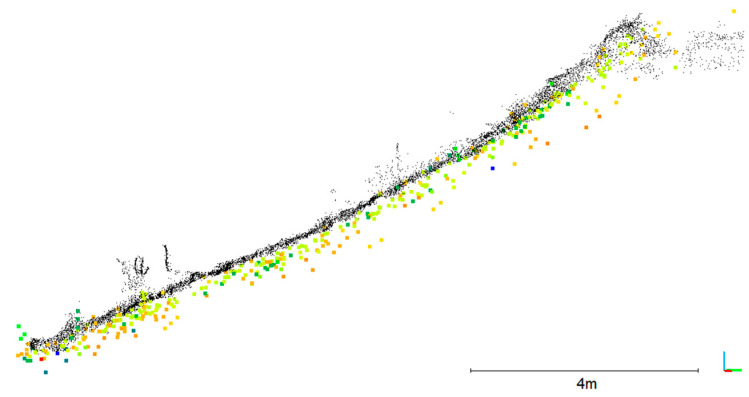
Cross section of mobile lidar point cloud (colorized according to UWV position) and TLS point cloud (black).

**Figure 16:**
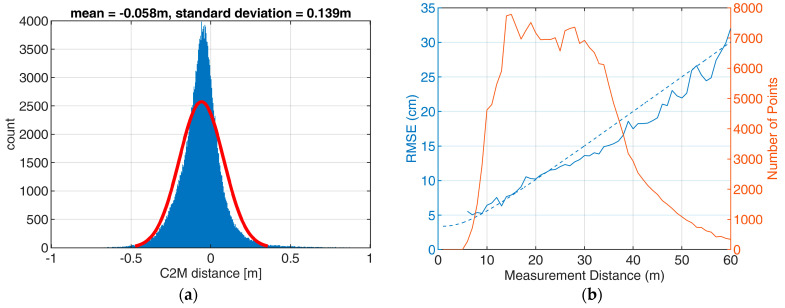
Mobile lidar point cloud to TLS mesh distance. (**a**) Histogram of cloud-to-mesh (C2M) distances for area of interest with a normal distribution fitted to the data (red line, parameters in figure title). (**b**) Distance-dependent theoretical (dashed) vs observed (solid) RMSE and numbers of points in each distance (red).

The area of interest, the railway embankment, has been used for an accuracy analysis. Therefore, this area was clipped from the oriented mobile lidar point cloud, resulting in a cloud of 218,000 points. This point cloud has been compared to the TLS reference that has 2.5 million points in that area. First, a mesh has been calculated from the reference point cloud, which was then used for a point-to-mesh distance calculation using CloudCompare. Figure 14 shows the result of that comparison. A cross section is shown in Figure 15. Figure 16a shows the histogram of the cloud-to-mesh distances. Besides a mean difference of 6 cm, an RMSE of 14 cm was achieved. It can be observed that there is no local accuracy dependency in the point cloud.

Analyzing cross sections of the Velodyne point cloud reveals that there is a large overall noise resulting from the measurements from different UWV positions and orientations (Figure 15). It highlights, furthermore, that the points tend to lie behind the reference. This is also reflected by the decentering of the histogram (Figure 16a).

The estimations from Section 6.1 suggest that the accuracy depends on the measurement distance. Therefore, distance-dependent RMSEs are calculated. The observed point-to-reference differences are split in 1 m pieces:

(10) {D−0.5 m<d≤D+0.5 m}

for D∈Z along the measurement range. The theoretical standard deviations have been calculated for the same distances following Equations (8) and (9), using the values from Table 1. Figure 16b shows the observed and expected RMSEs.

## 7. Conclusions

This study shows the potential of camera-based lidar orientation determination for an uncrewed multisensor water vehicle. GNSS and IMU depict the standard solution for the determination of position and orientation parameters of mobile mapping lidar systems. Differential GNSS offers the advantage of good overall global accuracy. The local accuracy may be improved by an IMU, which also offers a significantly higher temporal resolution. The major drawback of GNSS is signal loss in case of data acquisition in obstructed areas, as well as multi-path effects, which are, for instance, caused by GNSS signal double reflections on facades or water surfaces. The major drawback of IMUs is temporal drifts. The camera-based approach is primarily based on the automatic measurement of tie points in image sequences, thus not requiring a free line-of-sight to satellites—it will also work indoors, provided that there is sufficient texture in the image data for image matching. Sequential relative image orientation will also suffer from drift effects but, here, the camera-based approach offers the possibility of controlling drift effects by measuring control points (also called landmarks) in some of the images, thus geo-referencing the orientation data efficiently. Obviously, both methods—GNSS/IMU-based and camera-based determination of the position and orientation of a lidar sensor—may also be combined, but the goal of this paper was the analysis of the potential of the camera-based approach. A crucial requirement for the utilization of exterior camera orientations for the lidar points is the calibration of the relative orientation between the camera and the scanner coordinate systems. The presented strategy enables a fast single-shot calibration once the calibration field is established. This is more convenient when multiple systems have to be calibrated, compared to existing methods from the literature that use multiple shots of a planar test field. The resulting relative orientation can be determined very accurately with accuracies of <1 mm for the translations and <0.1° for the rotation angles. The calibration method could be applied for other multisensor systems as well.

**Figure 17:**
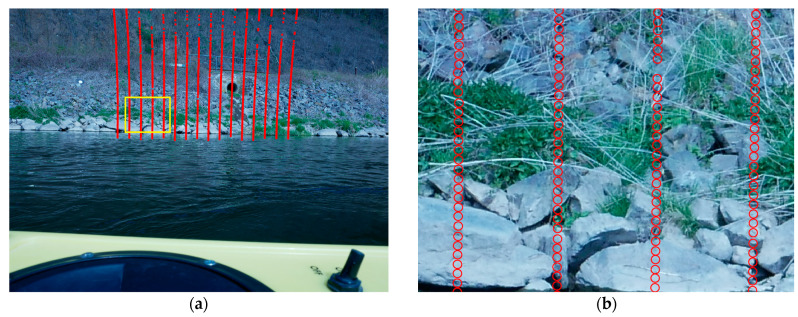
Footprints of mobile lidar. (**a**) Scan pattern of one Velodyne frame. (**b**) Detailed view (yellow box area in (**a**)) with measurement distances of approx. 30 m including footprint sizes depicted as circles.

The presented lidar transformation method was tested and analyzed based on a specific UWV configuration consisting of a Panasonic consumer camera and a Velodyne lidar. A theoretical accuracy analysis for this system showed that the largest errors occur from uncertainties in temporal synchronization between the camera and the scanner clocks. Using a camera with external triggering would largely eliminate this error. A practical experiment confirmed the theoretical analyses in terms of RMSE. It even performed superior to the expected RMSE. This could be caused by a bias of the cloud-to-cloud comparison. An inaccurate mobile lidar point is likely to be close to another TLS point, which it will be compared to. Additionally to the RMSE, a systematic offset of 5 cm was observed. This offset could be corrected by a registration of mobile and reference point clouds. Reasons for this behavior might be the different measurement behavior of vegetation by TLS and mobile lidar. Another influence is the inclination of the riverbank in combination with the laser footprint. The Velodyne lidar has a laser beam divergence of 3 mrad, resulting in a footprint with a diameter of 9 cm in 30 m distance which is 10 times the size of the TLS spot. Figure 17 suggests that this results in mixed signals.

While there are some drawbacks in the accuracy of the tested system, it has been shown that the method can be used as a valid positioning option. In a multisensor set-up, it can serve as a fallback option if other positioning systems fail. Furthermore, it can be used as an approximation, which is needed for other methods like lidar-based SLAM methods.

## Figures and Tables

**Table 1:** Standard deviations ($s_{v_{i}}$) of input parameters and their effect on the 3D point standard deviation in mm for world coordinates s3Dwcs in 5, 10, 25, and 50 m distance.

Name	$s_{v_{i}}$	s3Dwcs (mm)			
		5 m	10 m	25 m	50 m
ωccswcs (°)	0.078	5.7	11.3	28.0	55.8
ϕccswcs(°)	0.004	0.3	0.6	1.5	2.9
κccswcs(°)	0.078	5.6	11.0	27.4	54.7
Xccswcs(mm)	5.1	5.1	5.1	5.1	5.1
Yccswcs(mm)	5.4	5.4	5.4	5.4	5.4
Zccswcs(mm)	5.1	5.1	5.1	5.1	5.1
Δt(s)	0.023	21.1	42.5	112	229
ωscsccs(°)	0.067	4.7	9.4	23.4	46.8
ϕscsccs(°)	0.035	2.6	5.2	13.0	25.9
κscsccs(°)	0.015	1.0	2.1	5.2	10.5
Xscsccs(mm)	1.0	1.0	1.0	1.0	1.0
Yscsccs(mm)	0.4	0.4	0.4	0.4	0.4
Zscsccs(mm)	0.2	0.2	0.2	0.2	0.2
Xscs(mm)	17.3	17.3	17.3	17.3	17.3
Yscs(mm)	17.3	17.3	17.3	17.3	17.3
Zscs(mm)	17.3	17.3	17.3	17.3	17.3
$3D_{wcs}$		39	56	126	250

## Data Availability

The data presented in this study are available on request from the corresponding author. The data are not publicly available due to their large size.
